# Supplementary material for: Computational Study of the Kinetics and Mechanisms of Gas-Phase Decomposition of N-Diacetamides Using Density Functional Theory
Source: Molecules. 2024 Aug 13;29(16):3833. doi: 10.3390/molecules29163833 (PMC11356786; doi:10.3390/molecules29163833)
Supplement: Supplementary file 1 [file molecules-29-03833-s001.zip › molecules-3133493-SI.pdf]

Computational Study of the Kinetics and Mechanisms of Gas-Phase Decomposition of N-Diacetamides Using Density Functional Theory

Oswaldo Luis Gabidia Torres [1,*], Marcos Loroño [1,*] , Jose Luis Paz Rojas [2] , Cecilio Julio Alberto Garrido Schaeffer [3], Thais Cleofe Linares Fuentes [4] and Tania Cecilia Cordova Sintjago [5]

* Email: mloronog@unmsm.edu.pe

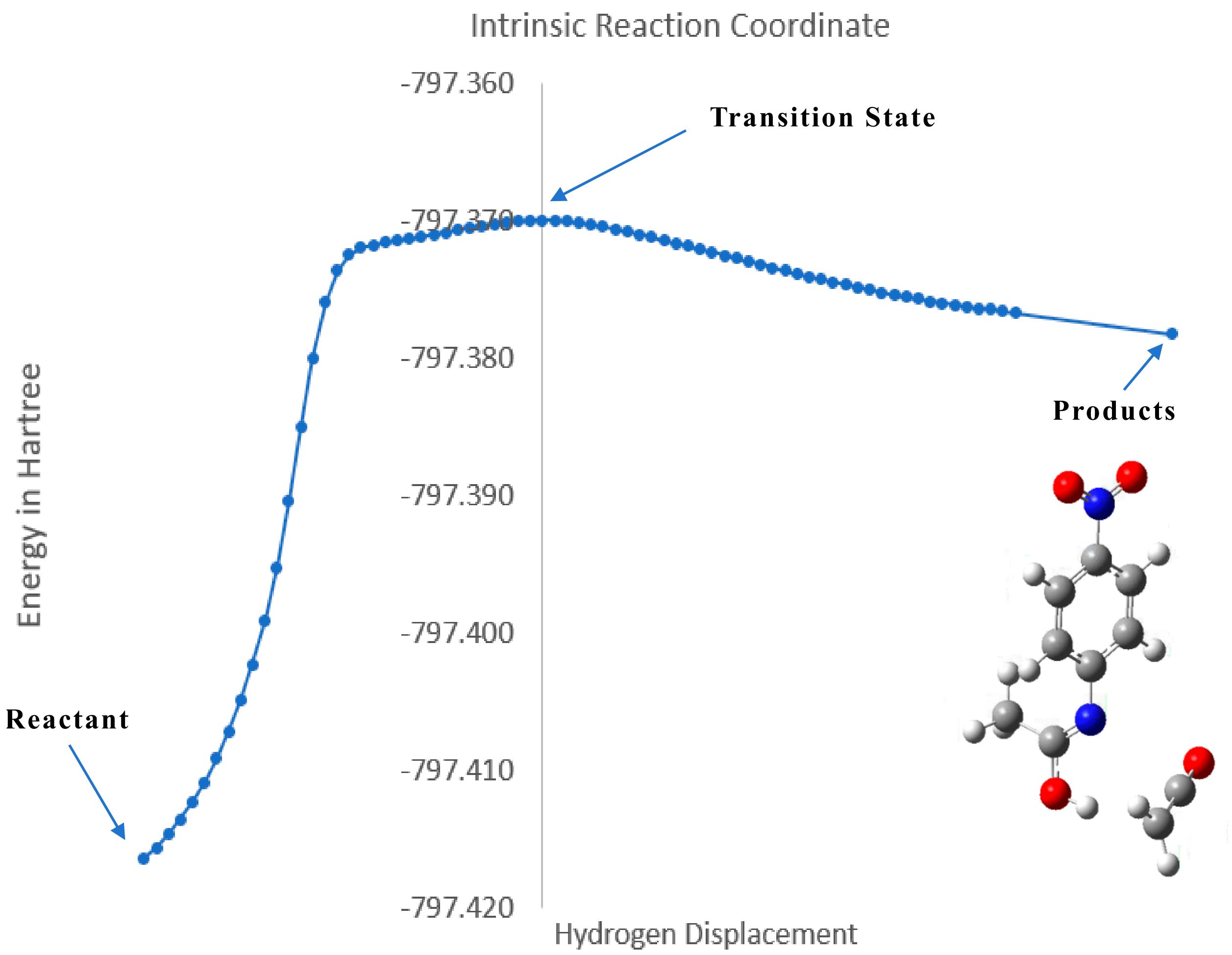

Figure S1. IRC calculation from reactant to products at theory level B3PW91/6-311+G(3df,2p) gd3bj

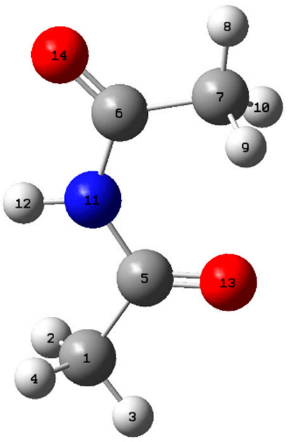

(A:monomer)

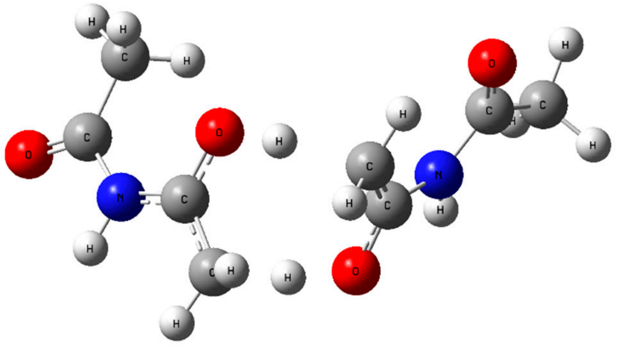

(B: dimer)

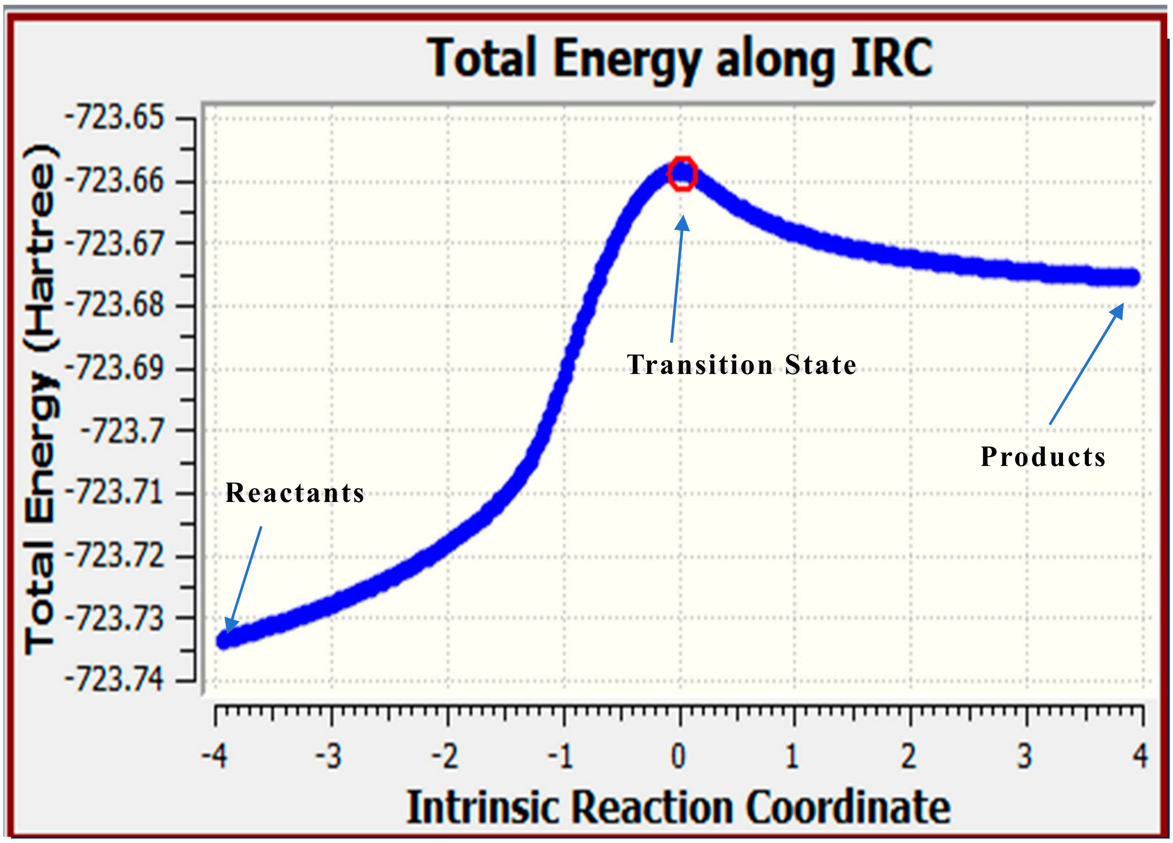

(C)

Figure S2. Dimer Proposal for the case of Diacetamide $NX(COCH_3)_2$ X = H. (A) monomer; (B) dimer, (C) IRC: intrinsic Reaction for the double hydrogen migration. Model is discussed in the main manuscript. Theory level: X3LYP/def2-TZVP.

Calculated thermodynamic data at level of theory X3LYP/def2-TZVP (A) Dimer, (B) monomer

(A): Dimer

Imaginary Freq	1	
Temperature	600.000	Kelvin
Pressure	1.00000	atm
Frequencies scaled by	1.0000	
Electronic Energy (EE)	-723.658572	Hartree
Zero-point Energy Correction	0.218177	Hartree
Thermal Correction to Energy	0.270751	Hartree
Thermal Correction to Enthalpy	0.272651	Hartree
Thermal Correction to Free Energy	0.097094	Hartree
EE + Zero-point Energy	-723.440395	Hartree
EE + Thermal Energy Correction	-723.387821	Hartree
EE + Thermal Enthalpy Correction	-723.385921	Hartree
EE + Thermal Free Energy Correction	-723.561478	Hartree
E (Thermal)	169.899	kcal/mol
Heat Capacity (Cv)	94.097	cal/mol-kelvin
Entropy (S)	183.607	cal/mol-kelvin

(B): monomer

Imaginary Freq	0	
Temperature	600.000	Kelvin
Pressure	1.00000	atm
Frequencies scaled by	1.0000	
Electronic Energy (EE)	-361.860259	Hartree
Zero-point Energy Correction	0.110767	Hartree
Thermal Correction to Energy	0.136759	Hartree
Thermal Correction to Enthalpy	0.138660	Hartree
Thermal Correction to Free Energy	0.025388	Hartree
EE + Zero-point Energy	-361.749492	Hartree
EE + Thermal Energy Correction	-361.723500	Hartree
EE + Thermal Enthalpy Correction	-361.721600	Hartree
EE + Thermal Free Energy Correction	-361.834871	Hartree
E (Thermal)	85.818	kcal/mol
Heat Capacity (Cv)	44.487	cal/mol-kelvin
Entropy (S)	118.464	cal/mol-kelvin

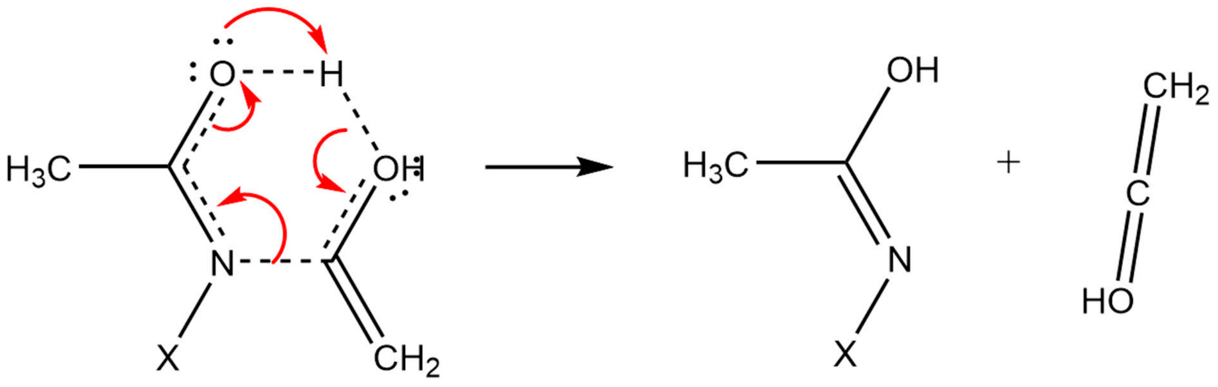

Scheme SS1. Final step-path of the dimer formation Scheme 3 (main manuscript)

NBO Analysis

Table ST1. Wiberg Indexes for the Phenyl-diacetamide/LC-BLYP– def2-TZVP

Wiberg Indices for LC-BLYP DEF2TZVP								
BONDS	Reactant	TS	Product	δB	%Ev	δBav	(δB-δBav)/δB	Sincronicity
N11-C5	1.0544	1.63310	1.7404	0.8436	84.36	0.8461	0.0030	0.9585
C5-O12	1.7420	1.13650	1.0322	0.8531	85.31		0.0083	
O12-H9	0.0011	0.63740	0.7020	0.9078	90.78		0.0729	
H9-C7	0.9102	0.05860	0.0165	0.9529	95.29		0.1262	
C7-C6	1.0136	1.61700	1.7289	0.8436	84.36		0.0030	
C6-N11	1.0069	0.33180	0.0072	0.6753	67.53		0.2019	
							0.4153	

A

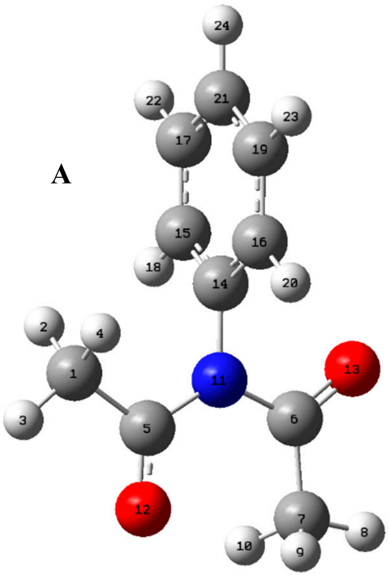

B

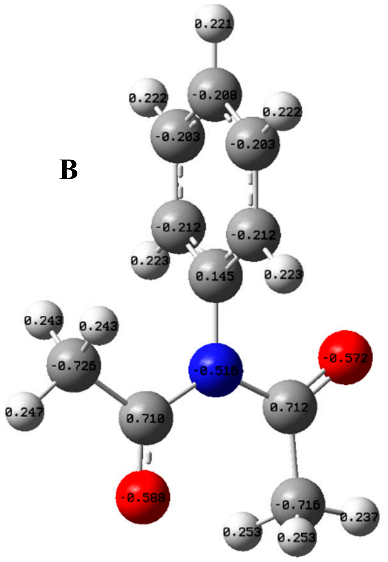

C

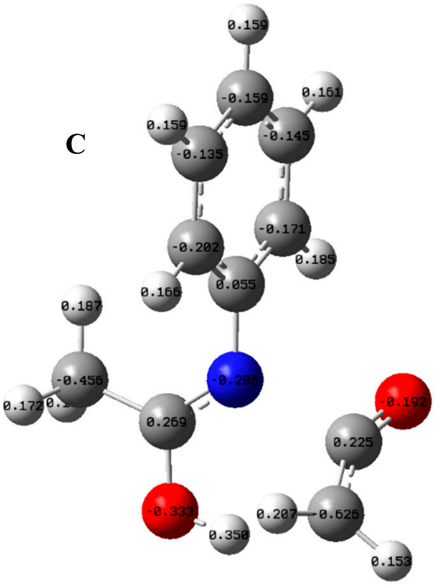

D

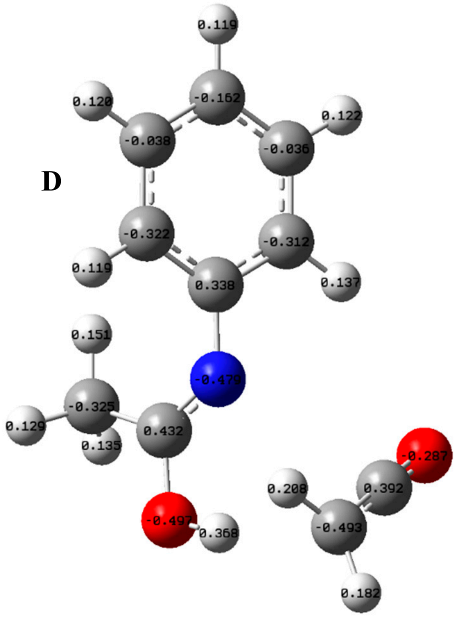

Figure S3. Phenyl-Diacetamide at the level of theory LC-BLYP– def2-TZVP. (A) Atomic label, (B) NBO charges (reactant), (B) NOB charges for the transition state (TS), y (D) NBO charges for the product.

Geometry input data for the Phenyl-Diacetamide at the level of theory LC-BLYP– def2-TZVP

Reactant

```
0 1
C    0.59440000    2.44957100   -0.00047700
H   -0.05219700    2.46602700    0.87423800
H    1.24110600    3.32027500   -0.00087600
H   -0.05213300    2.46517400   -0.87525900
C    1.47366100    1.23885000    0.00016100
C    1.46606100   -1.24363500    0.00053200
C    2.95232500   -1.32515700    0.00001100
H    3.20607100   -2.38054800   -0.00044400
H    3.37312400   -0.82552600   -0.86789900
H    3.37361900   -0.82613600    0.86802300
N    0.82650700    0.00905900    0.00033000
O    2.66973200    1.33884600   -0.00002700
O    0.78066500   -2.22715100   -0.00026000
C   -0.60450900   -0.04318100    0.00016500
C   -1.28684200   -0.07333900    1.19209900
C   -1.28657600   -0.07377400   -1.19189800
C   -2.66144500   -0.11422300    1.19166700
H   -0.73327400   -0.07187800    2.12198900
C   -2.66118800   -0.11467700   -1.19175400
H   -0.73281500   -0.07264900   -2.12167400
C   -3.34982300   -0.13180500   -0.00012000
H   -3.19905700   -0.13842300    2.12989400
H   -3.19858300   -0.13922000   -2.13009700
H   -4.43097000   -0.16584000   -0.00022700
```

Transition State

```
0 1
C   -0.75181100    2.49386000   -0.63189200
H   -0.98634900    3.24598800    0.11970000
H    0.32334800    2.41869700   -0.74867900
H   -1.20945500    2.81328000   -1.56598400
C   -1.35725100    1.20420100   -0.22674100
C   -1.70981800   -1.54559300    0.13311500
C   -2.61673500   -1.33844400    1.09171700
H   -2.47546900   -0.56999000    1.83005400
H   -3.00592700    0.35164600    0.09446100
H   -3.31698700   -2.13977500    1.27428300
N   -0.73505400    0.14384800    0.08116500
O   -2.67045700    1.23274600   -0.21113300
O   -1.26495900   -2.19951100   -0.72484200
C    0.66748700    0.04517300    0.07627500
C    1.39664400    0.48216400    1.16250900
C    1.30653400   -0.54737400   -0.99153700
C    2.76486400    0.34921900    1.16826900
H    0.88015200    0.92273800    2.00524100
C    2.67610900   -0.68095100   -0.97635500
H    0.71868900   -0.91130800   -1.82208900
C    3.40848500   -0.23174000    0.09845500
H    3.33351700    0.69791100    2.01997900
H    3.17549600   -1.14566500   -1.81596900
H    4.48464100   -0.33942900    0.10576600
```

Product

```
0 1
C    -0.12875100    2.56146500   -0.89367000
H    -0.17309700    3.45766800   -0.27680600
H     0.90718100    2.28141700   -1.05061900
H    -0.59825900    2.79943000   -1.84609000
C    -0.89693200    1.47418500   -0.23788900
C    -2.78440800   -1.54100000    0.13632200
C    -3.26477400   -0.95443900    1.19692700
H    -2.56909900   -0.54289300    1.90955500
H    -2.65961700    1.01390000    0.25295100
H    -4.31949300   -1.03399900    1.40390700
N    -0.48226600    0.37402300    0.19932700
O    -2.19492400    1.77024000   -0.14983100
O    -2.39513800   -2.05192500   -0.81079800
C     0.86498900    0.00601100    0.14748000
C     1.80384400    0.59527000    0.97124600
C     1.25558200   -1.01400500   -0.69878500
C     3.11688900    0.18516600    0.93151800
H     1.49210000    1.37599100    1.65300800
C     2.56951300   -1.41583100   -0.73751400
H     0.50974100   -1.48790400   -1.32336900
C     3.50747500   -0.81881600    0.07573200
H     3.84279700    0.65582500    1.58167800
H     2.86412600   -2.21120000   -1.40957600
H     4.53941900   -1.14094300    0.04690700
```

Table ST2. Wiberg indexes for the Diacetamide / B3PW91 – d3bj – def2-TZVP – First Stage

Wiberg Indices: B3Pw91 - gd3bj - Def2-TZVP								
BONDS	Reactant	TS	Producto	δB	%Ev	δBav	(δB-δBav)/δB	Sincronicity
C6-O13	0.0119	0.4495	0.9206	0.4816	48.16	0.566	0.1491	0.9503
O13-C5	1.7512	1.2962	0.9849	0.5938	59.38		0.0491	
C5-N11	1.0941	1.5438	1.8650	0.5833	58.33		0.0306	
N11-C6	1.0564	0.4229	0.0100	0.6054	60.54		0.0696	
							0.2984	

A B

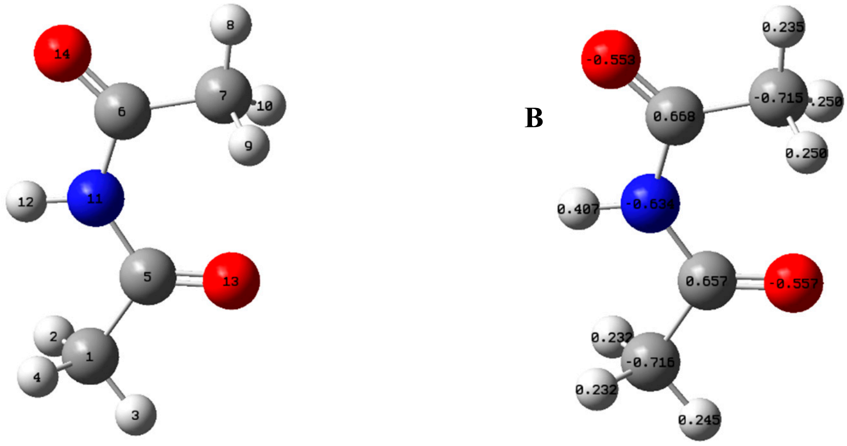

Figure S4. Diacetamide at the level of theory B3PW91-d3bj-def2-TZVP. (A) Atomic label, (B) NBO charges (reactant)

C

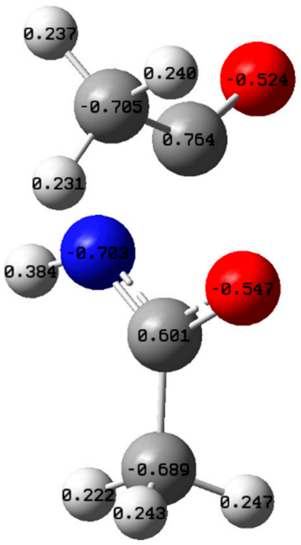

D

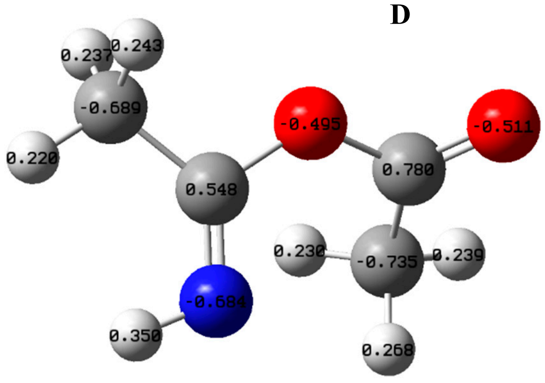

Figure S4. cont. Diacetamide at the level of theory B3PW91-d3bj-Def2-TZVP. (C) NBO charges (Transition State), (D) NBO charges (product)

Geometry input data for the Diacetamide at the level of theory B3PW91-d3bj–def2-TZVP

Reactant

0 1

C	2.46159600	-0.65021100	-0.00005100
H	2.51131500	-1.29214600	-0.88288800
H	3.30771700	0.03159000	-0.00031700
H	2.51164800	-1.29184800	0.88298600
C	1.18522400	0.15445900	0.00009100
C	-1.30737300	-0.27568600	0.00012500
C	-1.65911700	1.17742400	-0.00004700
H	-2.74340800	1.25858200	-0.00020900
H	-1.23366200	1.67653500	0.87097400
H	-1.23341400	1.67641600	-0.87101600
N	0.04534700	-0.63297600	0.00008900
H	0.17048600	-1.63534500	0.00005000
O	1.16470200	1.36207800	0.00000500
O	-2.12596400	-1.16568600	-0.00011900

Transition State

0 1

C	2.54301500	0.09568600	-0.10823400
H	2.91591200	-0.73960700	-0.70310600
H	2.78793000	1.01439500	-0.64407900
H	3.03622000	0.09876700	0.86235100
C	1.06376100	-0.02030400	0.02260900
C	-1.14180100	-0.20091800	-0.01065600
C	-1.72503300	1.17078000	-0.00588700
H	-2.38881700	1.25504700	0.85590100
H	-0.95839700	1.93786100	0.03926600
H	-2.31504600	1.28678100	-0.91681800
N	0.36399400	-0.08962700	1.10619100
H	0.76791700	-0.08561200	2.03198100
O	0.32016100	-0.05418400	-1.02226100
O	-1.67432700	-1.24727900	-0.05971800

Product

```
0 1
C          -2.35480000   -0.61988400    0.37301700
H          -2.45162600   -1.58819500   -0.12139000
H          -2.25840100   -0.81164800    1.44384000
H          -3.25000400   -0.02848400    0.19005200
C          -1.13540900    0.08342700   -0.12905900
C           1.26651700   -0.25898100   -0.00345100
C           1.54462000    1.09433600    0.57127300
H           2.57234700    1.09724200    0.92932700
H           1.42596800    1.84432100   -0.20995300
H           0.85238900    1.34926300    1.37229500
N          -1.03869800    1.21617400   -0.67301400
H          -1.94809600    1.66360400   -0.73032400
O          -0.04478900   -0.71727200    0.03025200
O           2.09513100   -1.01181700   -0.40943000
```

Table ST3. Wiberg indexes for the Diacetamide / B3PW91 – d3bj – def2-TZVP – Second Stage

Wiberg Indices: B3Pw91 - gd3bj - Def2-TZVP								
Bonds	Reactant	TS	Producto	δB	%Ev	δBav	(δB-δBav)/δB	Sincronicity
C5-O13	0.9849	1.2536	1.6721	0.3910	39.10	0.4098	0.0459	0.7829
O13-C6	0.9206	0.5981	0.0028	0.3514	35.14		0.1425	
C6-C7	1.0111	1.3253	1.7761	0.4107	41.07		0.0022	
C7-H9	0.8978	0.3348	0.0120	0.6356	63.56		0.5510	
H9-N11	0.0013	0.4511	0.7992	0.5637	56.37		0.3755	
N11-C5	1.8650	1.5519	1.2206	0.4859	48.59		0.1857	
							1.3028	

C

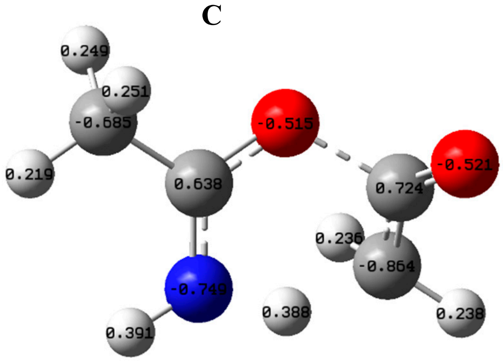

D

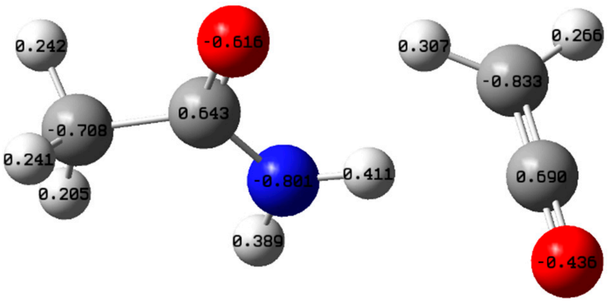

Figure S5. Diacetamide at the level of theory B3PW91-d3bj-def2-TZVP. (A) NBO charges (Transition State), (C) NBO charges (product)

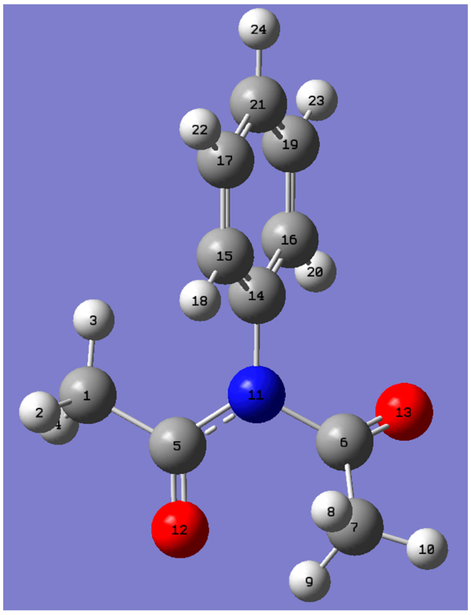

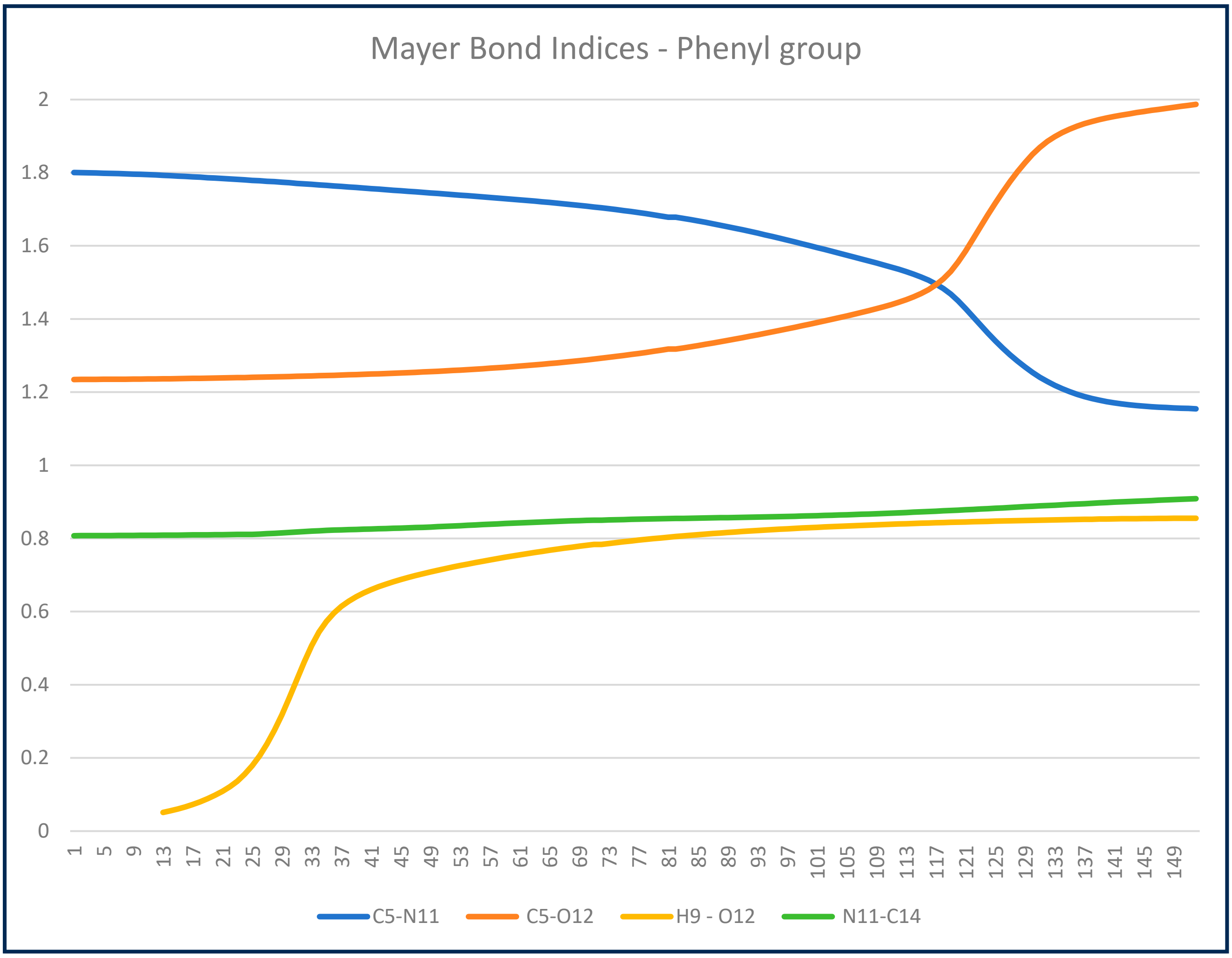

Figure S6. Mayer Bond order Analysis for Phenyl-diacetamide at the level of theory LC-BLYP– def2-TZVP data from an IRC calculation

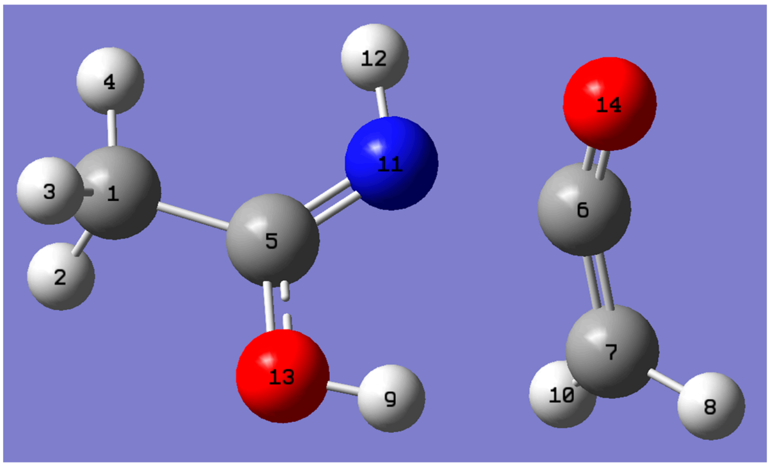

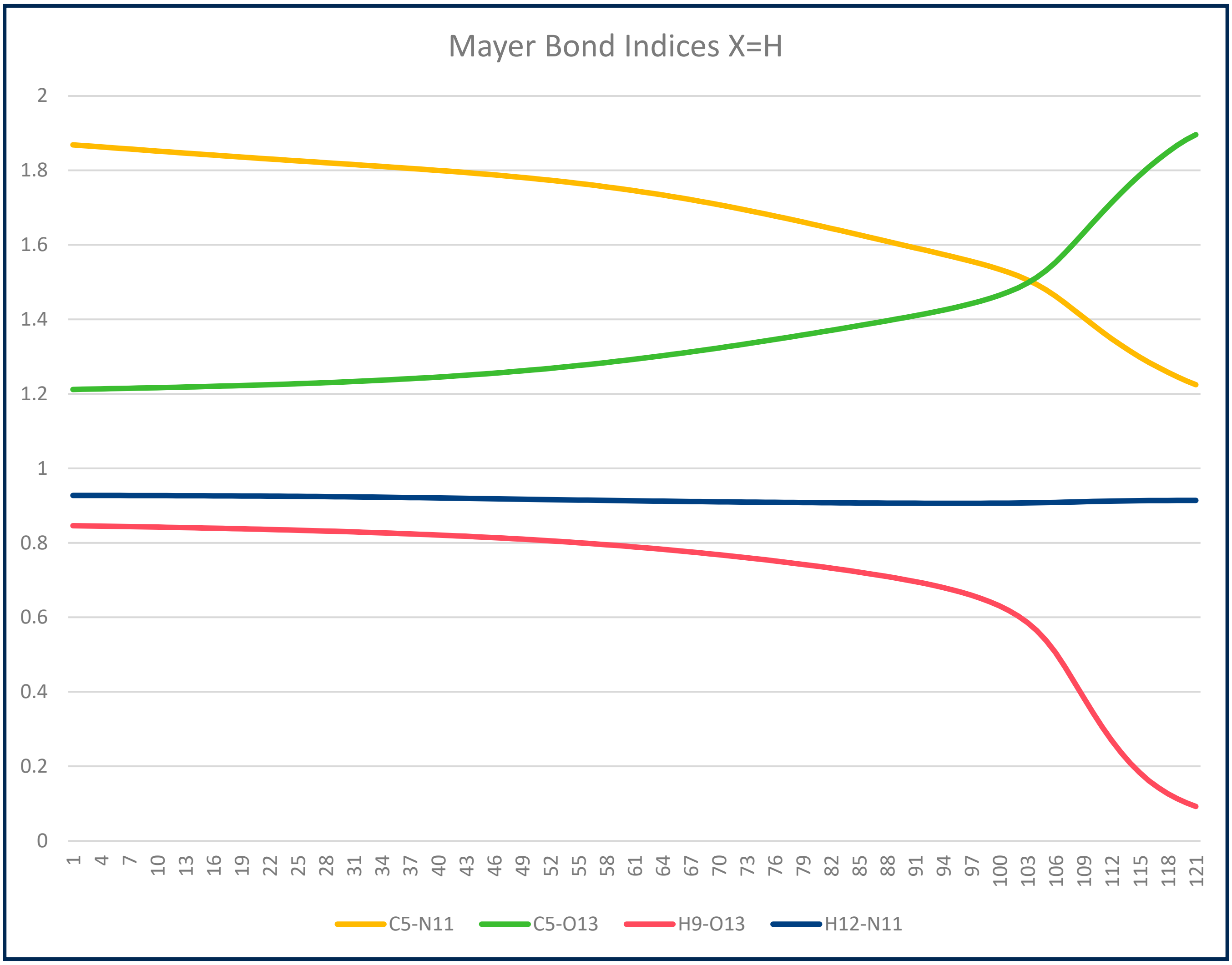

Figure S7. Mayer Bond order Analysis for Diacetamide at the level of theory LC-BLYP– def2-TZVP data from an IRC calculation

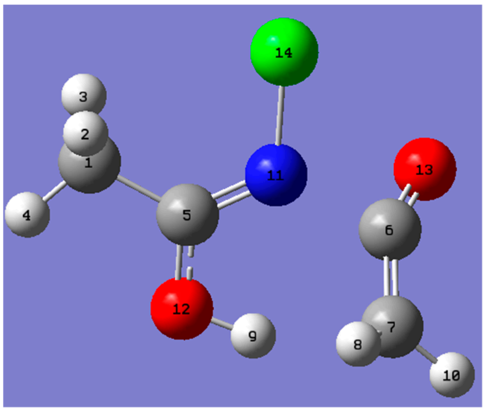

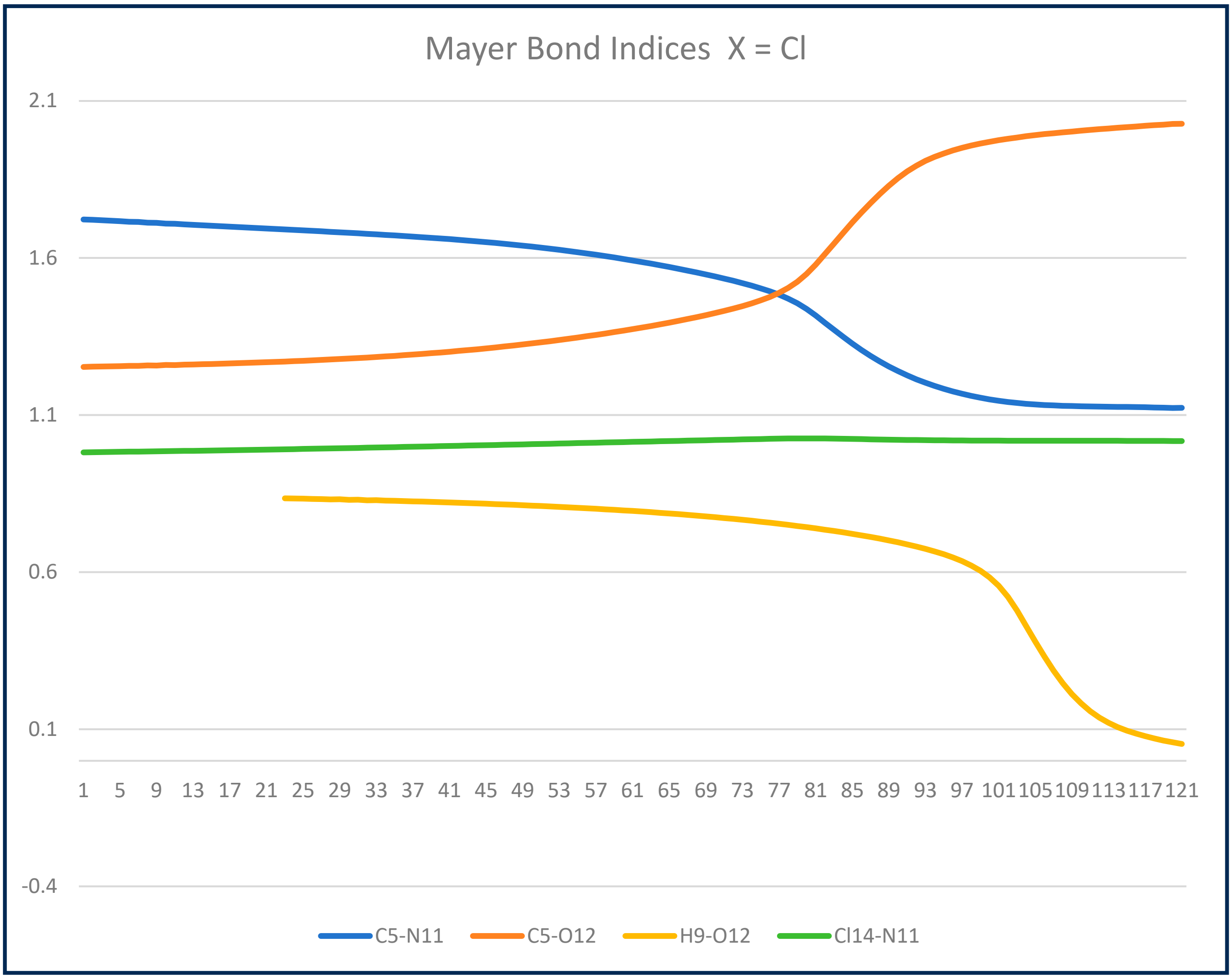

Figure S8. Mayer Bond order Analysis for Chloro-diacetamide at the level of theory LC-BLYP– def2-TZVP data from an IRC calculation

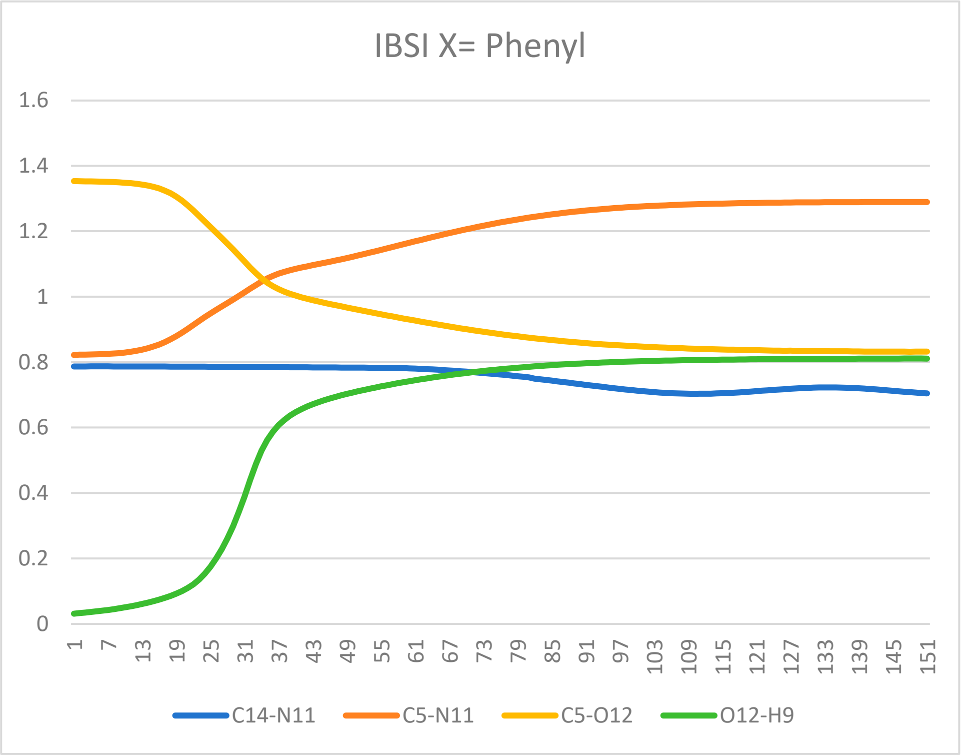

Figure S9. IBSI diagrams for the Phenyl-Diacetamide at the level of theory LC-BLYP– def2-TZVP data from an IRC calculation

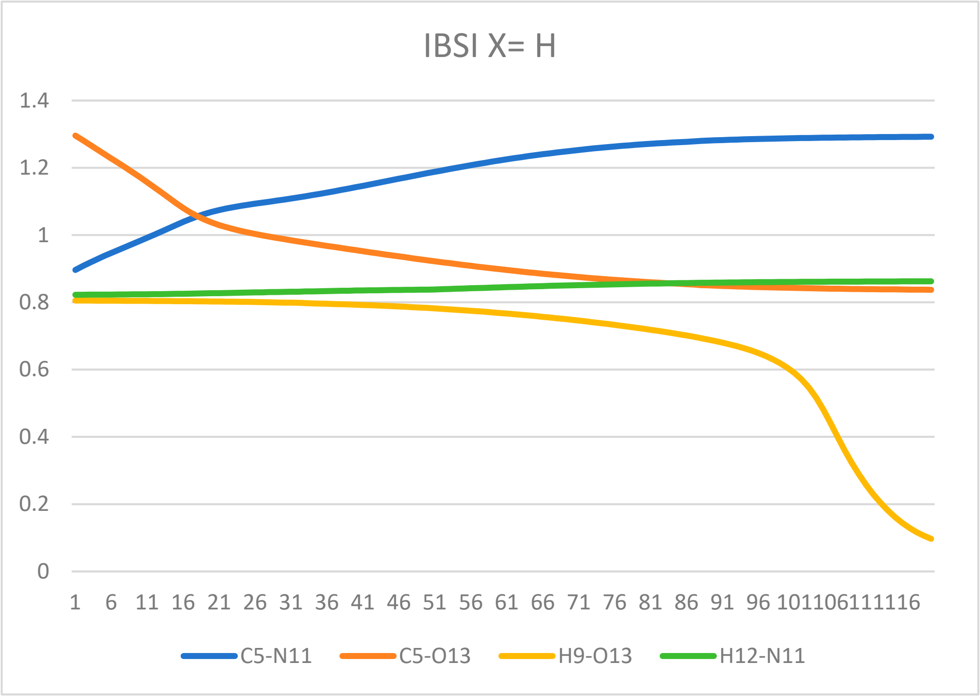

Figure S10. IBSI diagrams for the Diacetamide at the level of theory LC-BLYP– def2-TZVP data from an IRC calculation

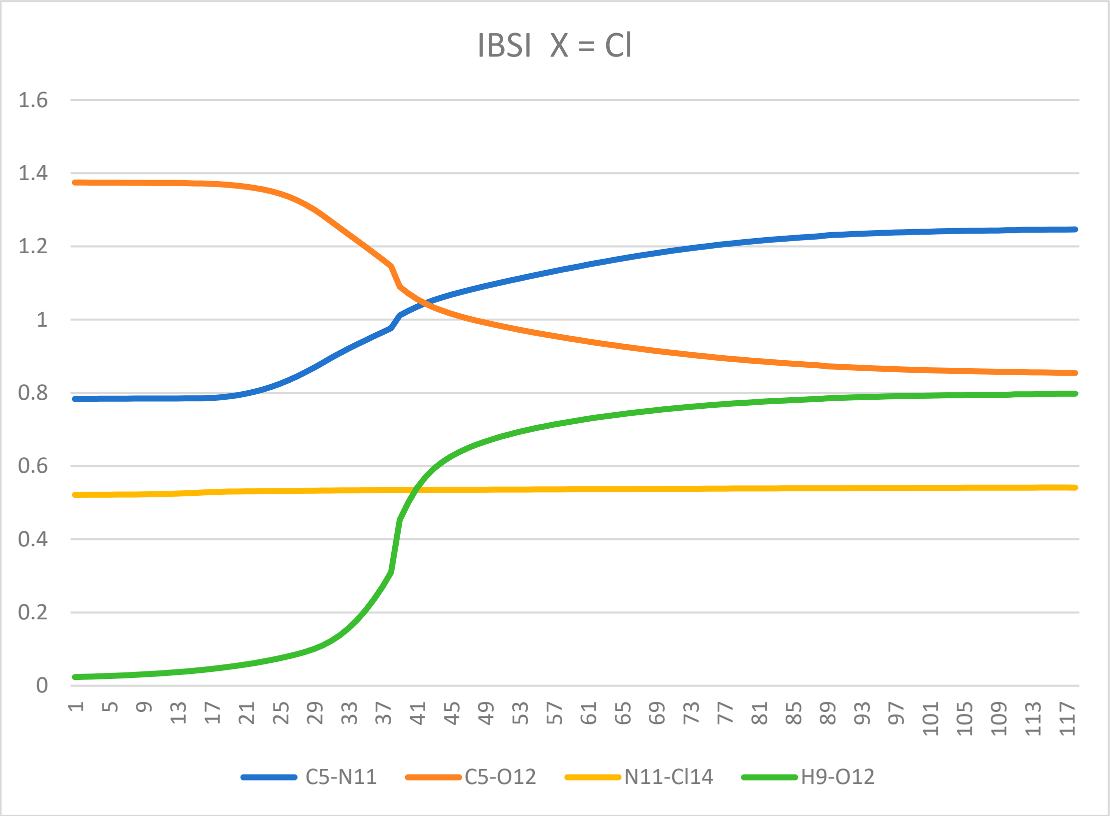

Figure S11. IBSI diagrams for the Chloro-Diacetamide at the level of theory LC-BLYP– def2-TZVP data from an IRC calculation
